# Supplementary material for: From sequence to enzyme mechanism using multi-label machine learning
Source: BMC Bioinformatics. 2014 May 19;15:150. doi: 10.1186/1471-2105-15-150 (PMC4229970; doi:10.1186/1471-2105-15-150)
Supplement: Additional file 2 — Java code of ml2db. Additional file ml2db_code.tar.gz contains the Java source code to run the multi-label machine learning experiments and save the results to database. The code’s Javadoc is included. [file 1471-2105-15-150-S2.zip › additional file 2/ml2db/ml2db/doc/mulan/classifier/neural/class-use/DataPair.html]

Uses of Class mulan.classifier.neural.DataPair


JavaScript is disabled on your browser.


- Overview
- Package
- Class
- Use
- Tree
- Deprecated
- Index
- Help

- Prev
- Next

- Frames
- No Frames

- All Classes

## Uses of Class mulan.classifier.neural.DataPair

- Packages that use DataPair

  | Package | Description |
  | --- | --- |
  |  |  |
  | --- | --- |
  | mulan.classifier.neural |  |
- - ### Uses of DataPair in mulan.classifier.neural

    Methods in mulan.classifier.neural that return types with arguments of type DataPair

    | Modifier and Type | Method and Description |
    | --- | --- |
    |  |  |
    | --- | --- |
    | `static java.util.List<DataPair>` | DataPair.`createDataPairs(MultiLabelInstances mlDataSet, boolean bipolarOutput)` Creates a `DataPair` representation for each `Instance` contained in `MultiLabelInstances` data set. |

    Methods in mulan.classifier.neural with parameters of type DataPair

    | Modifier and Type | Method and Description |
    | --- | --- |
    |  |  |
    | --- | --- |
    | `double` | MMPUpdateRuleBase.`process(DataPair example, java.util.Map<java.lang.String,java.lang.Object> params)` |
    | `double` | ModelUpdateRule.`process(DataPair example, java.util.Map<java.lang.String,java.lang.Object> params)` Process the training example and performs a model update when suitable. |

- Overview
- Package
- Class
- Use
- Tree
- Deprecated
- Index
- Help

- Prev
- Next

- Frames
- No Frames

- All Classes
